# Study on pharmacokinetic and tissue distribution of hyperin, astragalin, kaempferol-3-O-β-D-glucuronide from rats with multiple administrations of Semen Cuscutae processed with salt solution with effect of treating recurrent spontaneous abortion

**DOI:** 10.3389/fphar.2024.1440810

**Published:** 2024-09-16

**Authors:** Zhitong Yang, Kaiwen Chen, Yuting Zhang, Baiyang Xu, Yu Huang, Xue Zhang, Zilu Liu, Tongsheng Wang, Deling Wu, Tangyi Peng, Tulin Lu, Hao Cai, Xiaoli Wang

**Affiliations:** ^1^ Anhui Province Key Laboratory of Research, Development of Chinese Medicine, Anhui University of Chinese Medicine, Hefei, China; ^2^ Heritage Base of TCM Processing Technology of NATCM, Anhui University of Chinese Medicine, Hefei, China; ^3^ Anhui Province Key Laboratory of Traditional Chinese Medicine Decoction Pieces of New Manufacturing Technology, Hefei, China; ^4^ The First Affiliated Hospital of Anhui University of Chinese Medicine, Hefei, China; ^5^ School of Pharmacy, Nanjing University of Chinese Medicine, Nanjing, China

**Keywords:** recurrent spontaneous abortion, *Semen Cuscutae* processed with salt solution, correlation, UHPLC-MS/MS, active substance

## Abstract

**Introduction:**

*Semen Cuscutae* is a traditional Chinese medicine (TCM) that tonifies the kidneys and prevents miscarriage. According to Chinese medicine theory, kidney deficiency is one of the main causes of recurrent spontaneous abortion (RSA). The previous studies showed that raw product of *Semen Cuscutae* (SP) and *Semen Cuscutae* processed with salt solution (YP) have ameliorative effects on RSA, and that YP is superior to SP. However, the active components of YP to ameliorate RSA remain unclear and require further studies. The objective of this study is to investigate the active components of YP in ameliorating RSA.

**Methods:**

First, a rat model of RSA was established using hydroxyurea in combination with mifepristone. Aqueous decoction of YP was given by gavage to rats. Second, pregnant rats were sampled on days 5, 7, 9, 10 and 12 during the modelling period. The content of Hyperin (HY), astragalin (AS) and kaempferol-3-O-β-D-glucuronide (KA) in blood and liver, heart, spleen, lung and kidney tissues were detected by liquid chromatography-mass spectrometry (LC-MS). The pharmacodynamic indicators including progesterone (P), chorionic gonadotropin β (β-HCG), estradiol (E2), tumor necrosis factor-α (TFN-α), interleukin 4 (IL-4), and tryptophan (TRP) were measured by enzyme-linked immunosorbent assay (ELISA) Pearson's correlation analysis and grey relational analysis were used to establish the relationship between the pharmacodynamic indexes and chemical constituents.

**Results:**

The pharmacokinetic results showed that the area under curve (AUC) value of KA was the largest. The tissue distribution results showed that astragalin was widely distributed in liver, heart, spleen, lung and kidney in the RSA model rats, while HY was detected only in the uterus, and KA was detected only in the kidney. The pearson correlationl analysis showed that KA was significantly and positively correlated with the contents of E2, P, β-HCG and TRP. Both AS and HY were significantly negatively correlated with the content of TNF-α, respectively.

**Discussion:**

This study reveals the pharmacokinetics and tissue distribution of KA, AS and HY in rats with RSA. It was elucidated that all three were involved in the regulation of progesterone levels and immune function. It initially revealed the mechanism of action of YP in enhancing the improvement of RSA, and it provided a theoretical basis for the quality assessment of YP.

## Graphical Abstract

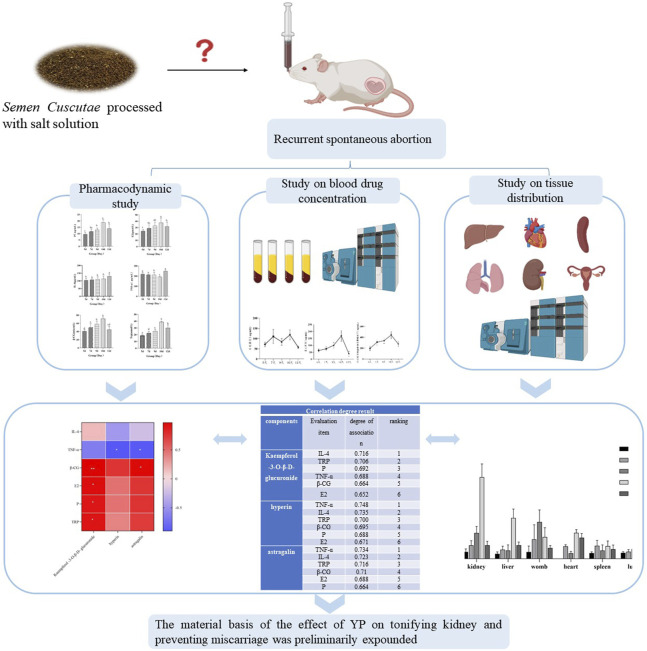

## Introduction

RSA refers to spontaneous abortion that occurs at least three times in the same woman. The incidence of RSA is approximately 1%–5% in women of child-bearing age (La et al., 2021). Patients with a history of RSA have a similar chance of miscarriage. RSA belongs to the category of “sliding fetus” in traditional Chinese medicine (TCM). It is one of the most common complications of pregnancy, with the characteristics of recurrent pregnancy and abortion. The causes of RSA are complex. In addition to genetic factors such as chromosome, luteal insufficiency and other endocrine factors, infection factors, immune anticoagulant antibody syndrome, pre-thrombotic state, internal reproductive organ anatomical abnormalities, and other influencing factors, the cause of RSA is unknown in 60%–70% of patients (Deng et al., 2022). The severity of RSA not only affects the physical and mental health of women but also makes the family and society unstable to a certain extent, which has gained the attention of scholars at home and abroad. The theory of “sliding abortion” points out that the essence of innateness is hidden in the kidney and the kidney andis responsible for reproduction. The completion of pregnancy lies in the consolidation of kidney qi (Xu et al., 2023a; Wang et al., 2020). Therefore, TCM, with the effect of kidney reinforcement, has unique advantages in improving RSA.

The frequency of use of 14,169 kinds of TCM prescriptions for recurrent abortion in the obstetrics and gynecology department of the First Affiliated Hospital of Anhui University of Chinese Medicine was analyzed and studied by our research group in the early stage. The results showed that YP was used most frequently. SP has significant effect on the application of tonifying the kidney and preventing fetus (Supplementary Figures S1, S2). SP was first recorded in Shennong’s Classic of Materia Medica and was listed as a quality medicine at the top grade (Xu et al., 2023b). Modern pharmacological studies have shown that SP can enhance immunity, improve reproductive function, result in anti-aging, and protect the liver (Zhang et al., 2024). The main components of SP mainly include flavonoids, glycosidic acids, alkaloids, and lignans (Liu et al., 2021). In addition, it was found that compared with SP, the total flavonoid content in YP increased (Xu et al., 2023c). As a traditional holy medicine for “tonifying kidney and preventing fetus,” the efficacy of YP has long been confirmed in clinical practice (Wang et al., 2021). It was recorded in Medical Zhongzhongcan Xilu written by Zhang Xichun’s that “SP has no roots and spreads on grass and trees, but the grass and trees are not lush. It is good to absorb the gasification of other things, which can be known by cultivation. The fetus is in its mother’s womb, and if it absorbs the gasification of its mother well, it will not fall down. In addition, both male and female reproduction rely on the kidney for strength. SP can strengthen the kidney qi, and the body with full kidney qi can nourish the foetus.” Previous research has shown that there were significant differences in the 10 components before and after salt processing with salt solution, and the flavonoids were generally increased after salt processing (Huang et al., 2022). However, the active substances of YP that treat RSA are still unclear.

*Semen Cuscutae* has more active components, with flavonoids being the most abundant. Modern pharmacology has shown that the total flavonoids of *Semen Cuscutae* have estrogenic effects, and KA, AS, and HY are the direct *in vivo* effectors of estrogenic effects of *Semen Cuscutae* (Sun et al., 2021). HY and KA, among others, can be used as quality markers for reproductive endocrine regulation, anti-inflammatory, immune enhancement, and inhibition of oxidative stress. The estrogenomimetic and anti-inflammatory effects of HY, AS, KA may reflect the disease state of recurrent miscarriage by regulating the hormone levels and expression of HY, AS, and KA. The estrogenomimetic and anti-inflammatory effects of HY, AS, and KA may reflect the disease state of recurrent miscarriage by regulating the hormone levels and expression of inflammatory factors in patients with RSA. Therefore, in this study, three components, HY, AS, and KA were selected as indicator components of *Semen Cuscutae*.

In the past decades, many experts and scholars have made remarkable achievements in the field of basic research on the medicinal effect of various materials. After the processing of TCM, the synergistic effect, phase system, and attenuation effect of the interaction between its components and the body is the embodiment of these comprehensive effects. Therefore, the *in vivo* study process is in line with the basic research mode of synergetic substances in TCM processing (Su et al., 2022). Pharmacokinetics study is helpful to reveal the pharmacodynamic substances of traditional Chinese medicine by studying the pharmacokinetic parameters of compounds, such as the area under the concentration–time curve (AUC), elimination half-life (t_1/2_), maximum concentration (C_max_), time to peak (T_max_), and tissue distribution. It is also important to elucidate the interactions of various components in the body. According to the “homing in meridian” theory of the role of TCM processing excipients, each drug has its favorable site of action in the human body. In other words, each drug or compound has a target organ in the body and exerts a therapeutic effect at that site. Therefore, it is necessary to carry out pharmacokinetic and tissue distribution studies of YP to elucidate its effective substances and mechanisms of action. In this study, a sensitive, reliable, and accurate ultra-performance liquid chromatography–tandem mass spectrometry (UHPLC-MS/MS) method was established for quantitative determination of the main components of YP in rat plasma and tissues, and it was successfully applied to the pharmacokinetics and tissue distribution of YP. The association model of composition change spectrum and effect index is an important tool to study the *in vivo* process of drugs. This model has the characteristics of accuracy and dynamic change, which plays a unique role in the study of the *in vivo* process of TCM.

This paper mainly uses the correlation model of “body composition–efficacy index” to initially explore the active substances of YP in the treatment of RSA, which will help in further enriching the scientific connotation of the theory of the role of Chinese medicine processing excipients, and it has important guiding significance for the improvement of Chinese medicine quality standards and processing technology.

## Materials and methods

### Materials and reagents

*Semen Cuscutae* herbal decoction pieces were purchased from Tongling Wada Chinese Medicine Drinking Tablets Company (Tongling, China) and confirmed as the dry matured seed of *Cuscuta australis* R. Br. or *Cuscuta chinensis* Lam. by Professor Liu Shoujin (Anhui University of Traditional Chinese Medicine). Previous studies have evaluated the quality of the characteristic profiles of S and YP (Supplementary Figures S3, S4).

Hydroxyurea tablets (Lot 1C0153D05) were purchased from Qilu Pharmaceutical Co., Ltd. (Jinan, China). Mifepristone tablets (Lot 05210302) were purchased from Jiulong Renwei Pharmaceutical Co., Ltd. (Wuhan, China). HY (Lot DJ0023-0020), AS (Lot DZ0001-0020), and KA (Lot DS0175-0005) were purchased from Chengdu Desite Biological Technology co., Ltd. (Chengdu, China), and three pure compounds were used as reference standards (purity ≥98%). Estradiol enzyme-linked immunosorbent assay (ELISA) kits were purchased from Shanghai Jianglai Biotechnology Co., Ltd. (Shanghai, China). Formic acid (R050195) was purchased from Sinopharm Chemical Reagent Co., Ltd. (Shanghai, China).

### Pharmacodynamic study

#### YP and YP water extract preparation

YP preparation: 2 g salt was dissolved in 10 mL water, and 100 g SP was stirred with the salt solution. After the salt solution was thoroughly absorbed, they were poured into a frying pan and heated on a mild fire for 4 min. The drugs were then removed from the container for cooling. YP water extract preparation: the YP was soaked in water for 30 min. Next, it was decocted twice with boiling water (1:10 and 1:8), 30 min for each, and then filtered. The filtrate was combined and concentrated to 10.8 g/kg.

#### Establishment, grouping, and morphological observation of the RSA model

Female and male SPF SD rats (200–220 g) were purchased from SPF (Beijing) Biotechnology Co., Ltd., and the animal license number was SCXK (Beijing) 2019-0010. The experimental rats were given free food and water for a week of adaptive feeding. The animal experiment involved in this experiment was examined and approved by the Research Ethics Committee of Anhui University of Traditional Chinese Medicine, and the approval number was AHUCM-rats-2022097.

The female and male rats were mated (mating ratio 2: 1). Vaginal smears of female rats were examined daily in the early morning hours. The pregnancy rate of rats was 50%. Day 0 of pregnancy is defined by the presence of a large number of sperm on the vaginal smear or the dropping of a vaginal plug (Supplementary Figure S5). Finally, 30 pregnant rats were randomly categorized into five groups (*n* = 6): 5-day group, 7-day group, 9-day group, 10-day group, and 12-day group. Hydroxyurea combined with mifepristone by gavage was used to establish the RSA model. The groups were given hydroxyurea (450 mg/kg) daily in the morning, and the 10.8 g/kg water extract of YP was administered in the afternoon for the groups. The 12-day group was given mifepristone on day 11 at a dose of 3.75 mg/kg (Wang et al., 2021). After 24 h of mifepristone administration, serum, blood plasma, and tissue samples of the liver, heart, spleen, lung, kidney, and uterus were obtained from each group of rats after being anesthetized by intraperitoneal injection with pentobarbital (30 mg/kg). After anesthetizing the rats, the surrounding fat and other tissues were stripped, and the uterus and kidney were weighed. Plasma and tissues (liver, heart, spleen, lung, kidney, and uterus) taken at 5, 7, 9, 10, and 12 days were used for pharmacokinetic and tissue distribution studies, respectively.

#### Calculation of organ index

During the experiment, the behavior of the rats was observed daily, and their body weight was recorded. The organ index of the liver, heart, spleen, lungs, kidneys, and uterus was calculated using the following formula: organ index = organ weight (g)/body weight (g) × 100%.

#### Biochemical index detection

On days 5, 7, 9, 10, and 12 of pregnancy, rats were anesthetized by intraperitoneal injection of 20% Ultane, and blood was collected from the abdominal aorta. After 2 h at room temperature, the sample was centrifuged for 3,500 r/min at low temperature for 10 min, the upper serum was separated, and the levels of serum IL-4, TNF-α, P, E2, and other indexes were detected by ELISA.

#### Statistical analysis

The levels of serum progesterone (P), estradiol (E2), tumor necrosis factor-α (TNF-α), interleukin-4 (IL-4), rat chorionic gonadotropin (β-CG), and tryptophan (TRP) were measured and calculated according to the instructions of the kit. GraphPad Prism software (version 8.0, GraphPad Software, United States) was used to statistically analyze the experimental data. The data in the experiment were finally expressed as $\bar{x}$ ± s. Significance analysis was performed using the one-way analysis of variance (ANOVA) and was used for statistical comparison. In this experiment, *p* < 0.05 was regarded as the threshold of significance.

### Pharmacokinetic study

#### YP and YP water extract preparation

The preparation method of YP and YP water extract was the same as that described in the part of “pharmacodynamic study/YP and YP water extract preparation” above.

#### Establishment, grouping, and morphological observation of the RSA model

The experiment of the establishment, grouping, and morphological observation of the RSA model was the same as that described in the part of “pharmacodynamic study” above.

#### Sample preparation

Rat tissue samples were homogenized with ice saline solution and centrifuged at 12,000 rpm for 15 min, and the supernatant was taken out.

In a 1.5-mL EP tube, 10 μL solution of IS was added to 100 μL of plasma samples, and 300 μL methanol was added to precipitate protein. The mixture was vortexed for 2 min and centrifuged at 12,000 rpm for 10 min at 4°C. The supernatant was transferred to a new EP tube and then dried under nitrogen at 35°C. The dried residue was re-dissolved with 100 μL methanol:water (50:50, v/v) solution. After being centrifuged at 12,000 rpm for 10 min at 4°C, a 10-μL aliquot of the supernatant was injected into LC–MS/MS for analysis.

#### Preparation of standards, calibration standards, and quality control samples

Appropriate amounts of KA, HY, and AS were accurately weighed and prepared with methanol with mass concentrations of 0.200 mg/mL, 0.200 mg/mL, and 0.204 mg/mL, respectively. Glipizide was dissolved in methanol and further diluted to 200 ng/mL to obtain the IS solution. Seven aliquots of 90 μL blank plasma were spiked with 10 μL of a mixed standard solution of KA, HY, and AS to yield calibration standards with concentrations in the range of 1–1,000 ng/mL KA, 1–1,000 ng/mL HY, and 1–1,000 ng/mL AS. Quality control (QC) samples were independently prepared in a similar manner to obtain three levels of samples: low, medium, and high concentrations for each analyte.

#### Chromatographic and mass spectrometry conditions

The chromatographic separation was performed on an ACQUITY UPLC®BEH C18 (2.1 × 100 mm, 1.7 μm) with the column temperature maintained at 35°C. Mobile phase A consisted of methanol and mobile phase B consisted of 0.1% formic acid. The flow rate was 0.3 mL/min, and the injection volume was 5 μL. The gradient elution procedure was as follows: 0–5 min, 45 %–80%A; 5–5.5 min, 80%–45%A; 5.5–6.0 min, 45%A.

**FIGURE 1:**
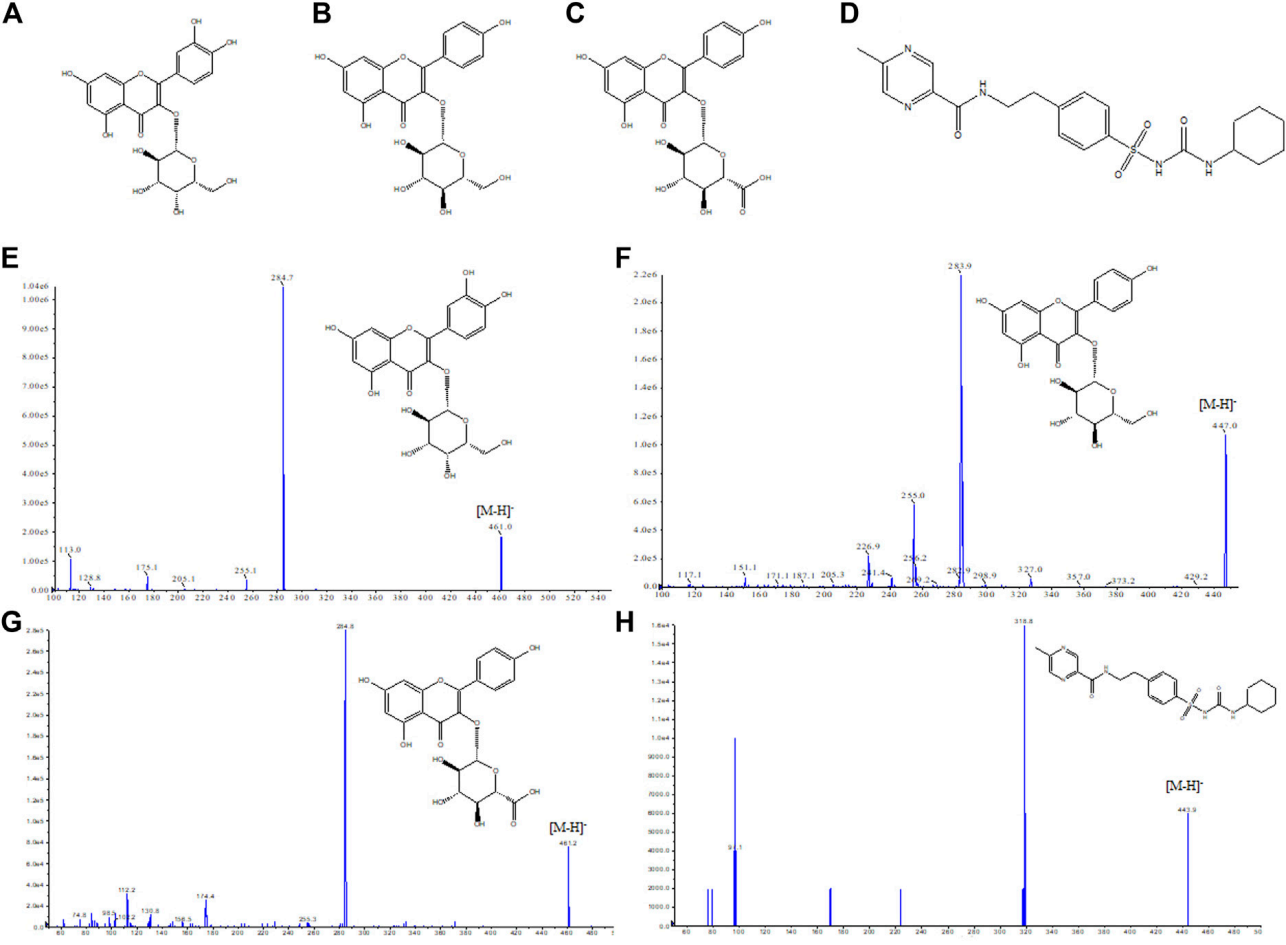
Chemical structure of three compounds and mass spectrum of each component to be detected and the internal standard compound. **(A)** Hyperin, **(B)** astragalin, **(C)** kaempferol-3-O-β-D-glucuronide, and **(D)** glipizide. **(E)** Mass Spectrometry of Hyperin, **(F)** Mass Spectrometry of astragalin, **(G)** Mass Spectrometry of kaempferol-3-O-β-D-glucuronide, **(H)** Mass Spectrometry of glipizide.

For mass spectrometry conditions, the analyte and IS were ionized in the ESI negative ion mode. The ion source temperature was 500°C, the ion spray voltage was –4,500 V, Gas1 was 50 psi, Gas2 was 50 psi, and the MRM mode for quantification of KA was m/z 461.0→284.7. The MRM mode for quantification of HY was m/z 463.2→300.0. The MRM mode for quantification of AS was m/z 447.1→283.9. The MRM mode for quantification of IS was m/z 444.1→319.0 (Figure 1).

#### Method validation

##### Selectivity validation

Blank plasma and kidney samples were collected for selectivity validation, and the blank samples were mixed with KA, AS, HY, and IS solutions for further testing. Rat plasma and kidney tissues 2 h after continued dosing of YP on the fifth day were collected to determine the KA, AS, and HY. All samples were processed according to the methods described in the part of “sample preparation.”

##### Linearity validation

The linearity of the standard curve was determined by plotting the peak area ratio (KA, AS, and HY/IS, *y*-axis) versus their concentration (*x*-axis) in plasma using a weighted linear least-squares regression model (1/x^2^). The samples were processed according to the methods described in the part of “sample preparation.”

##### Lower limit of quantitation validation

The lower limit of quantitation (LLOQ) was defined as the lowest concentration of three analytes that were precisely added to the blank plasma for detection. The samples were handled in the same manner as described in the part of “sample preparation.” The relative standard deviations (RSDs) of the concentrations of the three analytes in the samples were calculated.

##### Accuracy and precision validation

The intra-day and inter-day precision and accuracy of the three analytes in the rat plasma, liver, heart, spleen, lung, kidney, and uterus tissues were obtained by analyzing the low and high concentration QC samples in six replicates on day one and three consecutive days, respectively.

##### Extraction recovery and matrix effect validation

The extraction recovery of KA, AS, and HY was evaluated at low, middle, and high concentration levels by comparing the peak areas of the two analytes in the blank plasma-spiked samples with those of the post-extraction spiked samples. The matrix effect was measured at three concentration levels by comparing the peak areas of the analytes from the post-extraction spiked blank samples to those of the aliquots of methanol spiked with the same standard.

##### Stability validation

The stability of the analytes in the rat plasma, liver, heart, spleen, lung, kidney, and uterus tissues, including short-term and freeze–thaw stability, was evaluated at low and high concentration levels in six replicates under diverse conditions: 12 h at room temperature (short-term stability), 4 weeks at −20°C (long-term stability), and after three freeze–thaw cycles (freeze–thaw stability).

#### Statistical analysis

Data regarding KA, AS, and HY from plasma concentration–time curves were evaluated using DAS 2.0 software (Mathematical Pharmacology Professional Committee of China, Shanghai, China). Pharmacokinetic parameters were analyzed using SPSS 21.0 software.

#### Tissue distribution study

A study was conducted to study the distribution of KA, AS, and HY in the liver, heart, spleen, lung, kidney, and uterus tissues. The grouping, administration, and tissue sampling of rats were the same as previously mentioned in part of “pharmacokinetic study/establishment, grouping, and morphological observation of RSA model.”

### Gray relationship analysis of pharmacodynamics and pharmacokinetics

Gray correlation analysis (GCA) was performed using the data processing system SPSS software (version 23.0, IBM, United States) to establish the pharmacodynamics and pharmacokinetics relationship, that is, the relationship between the components and efficacy.

## Results

### The therapeutic effect of YP on RSA rats

#### Assessments of body weight and organ index

**FIGURE 2:**
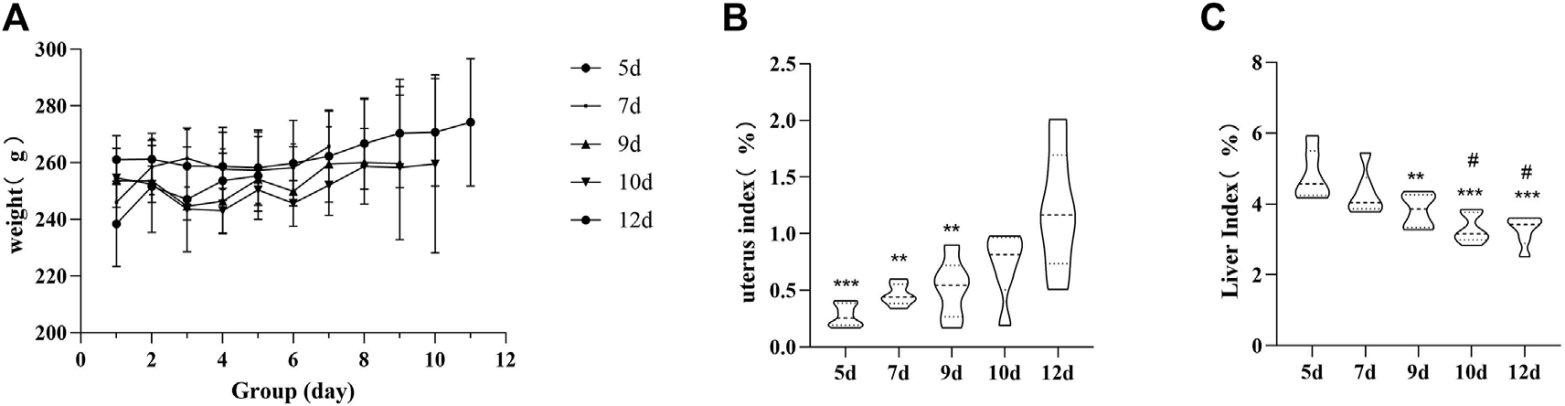
Rat body weight **(A)**, changes of uterine coefficient **(B)**, and changes of liver coefficient **(C)**. Uterus: comparison of 12-day group, ^##^ *p* < 0.05, ^△△^ *p* < 0.05, ***p < 0.001.

To explore the therapeutic effect of YP on RSA, we treated RSA rats with YP decoction at doses of 10.8 g/kg for 12 days. There was a slow increase in the body weight of pregnant rats in the 5-, 7-, 9-, 10-, and 12-day groups, and the body weight of rats in the 7-day group was significantly higher than that of rats in the first day of the gestation group (*p* < 0.01). The gradual increase in uterine coefficients and decrease in liver coefficients during the side modeling and administration of the drug suggest that YP has a protective effect on the fetus (Figures 2A–C).

#### Effects of YP on serum biochemical indicators

**FIGURE 3:**
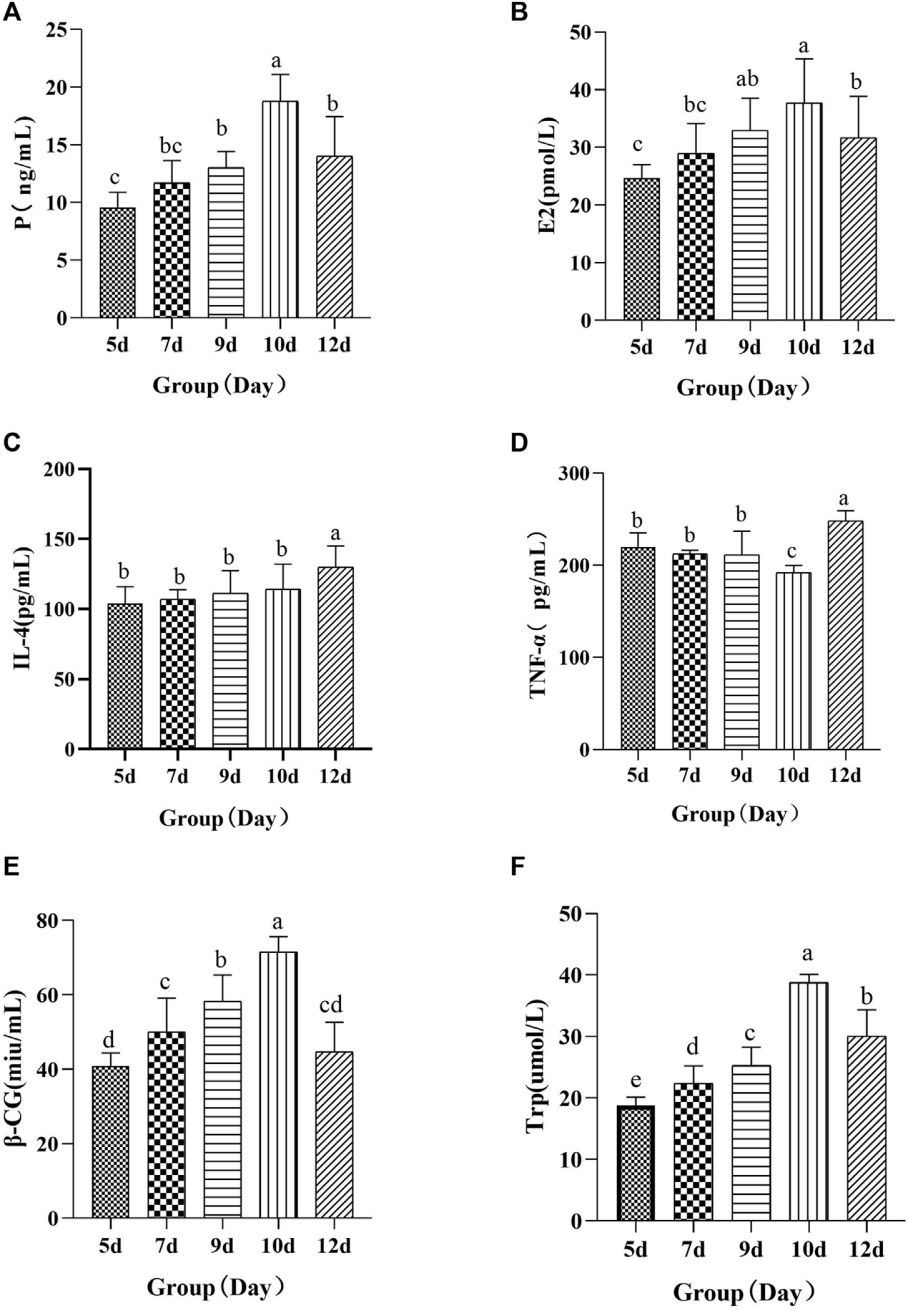
Changes of serum P **(A)**, E2 **(B)**, IL-4 **(C)**, TNF-α **(D)**, β-HCG **(E)**, and TRP **(F)**. Different letters in the figure indicate significant differences among the samples (*p* < 0.05).

The results of biochemical indicators showed that compared with the 5-day group, the serum concentrations of E2, P, β-HCG, IL-4, and TRP in the 10-day group increased significantly, whereas the concentration of TNF-α decreased significantly. These results showed that YP may cause improvement in rats with RSA by regulating E2, P, β-HCG, TRP, IL-4, and TNF-α. Compared with the 10-day group, the serum concentrations of E2, P, β-HCG, and TRP in the 12-day group decreased significantly, whereas the concentration of IL-4 and TNF-α increased significantly. This shows that the establishment of RSA is successful (Figures 3A–F).

### Pharmacokinetic study

#### Optimization of UHPLC-MS/MS

To obtain appropriate retention times, good separability, and symmetrical peak shapes, 0.1% formic acid aqueous solution (methanol) was chosen as a suitable mobile phase. According to the comprehensive consideration of the matrix effect and determination efficiency, the final optimized gradient elution program was as follows: 0–5 min, 45%–80% methanol; 5–5.5 min, 80%–45% methanol; 5.5–6.0 min, 45% methanol.

To optimize the MS spectra, the positive and negative ion modes of the first-order mass spectrometry of the analytes and IS solutions were investigated. It was found that the MS detection of HY, AS, and KA was sensitive in the negative ion mode, whereas IS responded in both the positive and negative ion modes. Therefore, the negative ion mode was selected for the determination, and the optimized MS parameters to achieve maximum responses are summarized in Table 1.

**TABLE 1:** Mass spectrometry conditions of three components and the internal standard.

Compound	Parent ion (m/z)	Daughter ion (m/z)	DP/V	CE/V	ESI
Kaempferol-3-O-β-D-glucuronide	461.00	284.80	−102.7	−27.93	—
Hyperin	463.20	299.60	−118.86	−36.13	—
Astragalin	447.10	283.80	−102.90	−34.07	—
Glipizide	444.10	319.00	−113.25	−27.12	—

#### Method validation

##### Specificity

**FIGURE 4:**
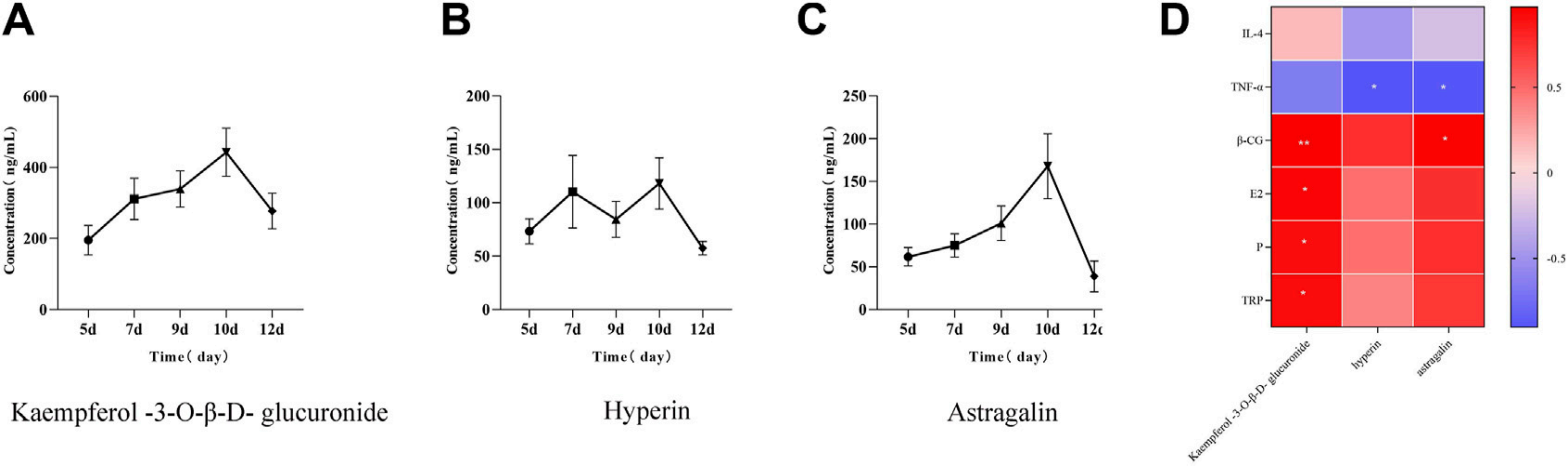
*In vivo* process and correlation analysis of three components in YP. **(A)** Blood concentration–time curve of KA, **(B)** blood concentration–time curve of HY, **(C)** blood concentration–time curve of AS, and **(D)** thermogram of correlation coefficient between blood components and pharmacodynamic indexes of YP.

The retention times of HY, AS, KA, and IS are shown in Figure 4. The chromatograms of the three analytes and IS in the blank plasma, blank plasma spiked with the analytes and IS, and plasma samples collected after oral administration of YP for 5 days 2 h were compared. The same sample treatments and comparisons were performed for kidney tissues. The results showed symmetric peaks of the analytes and IS with no endogenous substance interferences, stable baselines, and high specificity (Supplementary Figure S6).

##### Linearity and LLOQ

The standard curve and linear regression equation of the analytes that were established by detection of the plasma and kidney samples containing various concentrations of HY, AS, KA. HY, AS, KA, exhibited good linearity, with correlation coefficients (*R* ^2^) ≥ 0.99. The LLOQs of naringin and paeoniflorin are listed in Supplementary Table S1. The results showed that the method was suitable for the quantitative analysis of the two analytes and met the pharmacokinetic requirements.

##### Accuracy and precision

The results showed that the intra- and inter-day precisions of the three analytes were within 14.72%, and the accuracy ranged from −14.38% to 14.32% (Supplementary Table S2). All analysis values were within the accepted criteria, proving that the method was accurate and reliable for the target analytes.

##### Extraction recovery and matrix effect

The recovery of the analytes was greater than 90.87%, and the matrix effects were between 91.84% and 107.21%. The above results showed that the analysis of the three analytes in the plasma and kidney tissue had no obvious matrix effect (Supplementary Table S3).

##### Stability

The short-term stability and freeze–thaw stability of the analytes in the plasma and kidney tissue were evaluated and are shown in Supplementary Table S4. The results displayed RSD values less than 14.04%, indicating that the biological samples were stable during these stability tests.

### Pharmacokinetic evaluation

The validated LC–MS/MS method was successfully applied to the pharmacokinetic study of HY, AS, and KA in rat plasma after oral administration of the YP decoction. As depicted in Figure 4, double peaks appeared in the concentration–time curve of HY in RSA rats administered YP. The first peak concentration of HY appeared at 7 days, and the second peak concentration appeared at 10 days. In addition, the second peak concentration was much higher than the first one. Compared with the other two compounds, the area under the curve of KA is the largest (Figures 4A–C).

### Tissue distribution of the analytes in rat tissues

**FIGURE 5:**
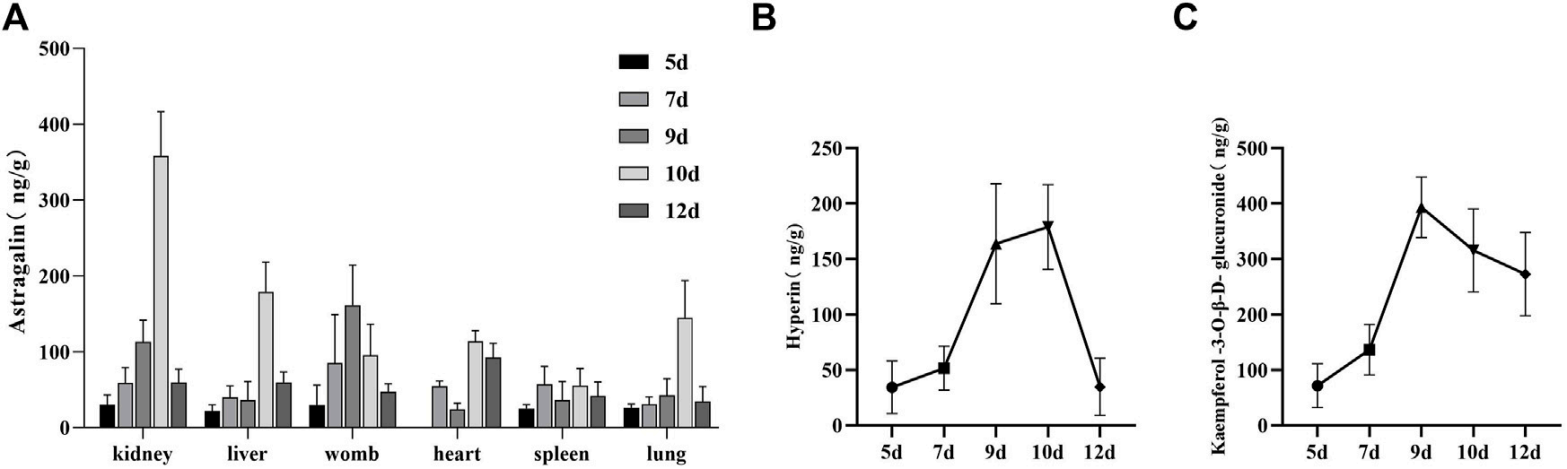
**(A)** Tissue distribution characteristics of AS, **(B)** tissue distribution characteristics of KA, and **(C)** tissue distribution characteristics of HY.

The tissue distribution of AS was extensive in rats, and they were examined in the liver, heart, spleen, lung, kidney, and womb tissues (Figures 5A–C). AS was detected in the heart tissue on the 7th day. The concentration of AS reached the maximum on the 9th day in womb tissue, and that of other tissues reached the maximum on the 10th day. Compared with the 10-day group, the concentration of AS in the liver, heart, lung, and uterus decreased rapidly in the 12-day group. HY was only detected in the womb, and the concentration reached the peak on the 10th day and decreased rapidly on the 12th day. KA was only detected in renal tissue, reaching the peak on the 9th day, and still having a high concentration on the 12th day.

### Correlation analysis

**FIGURE 6:**
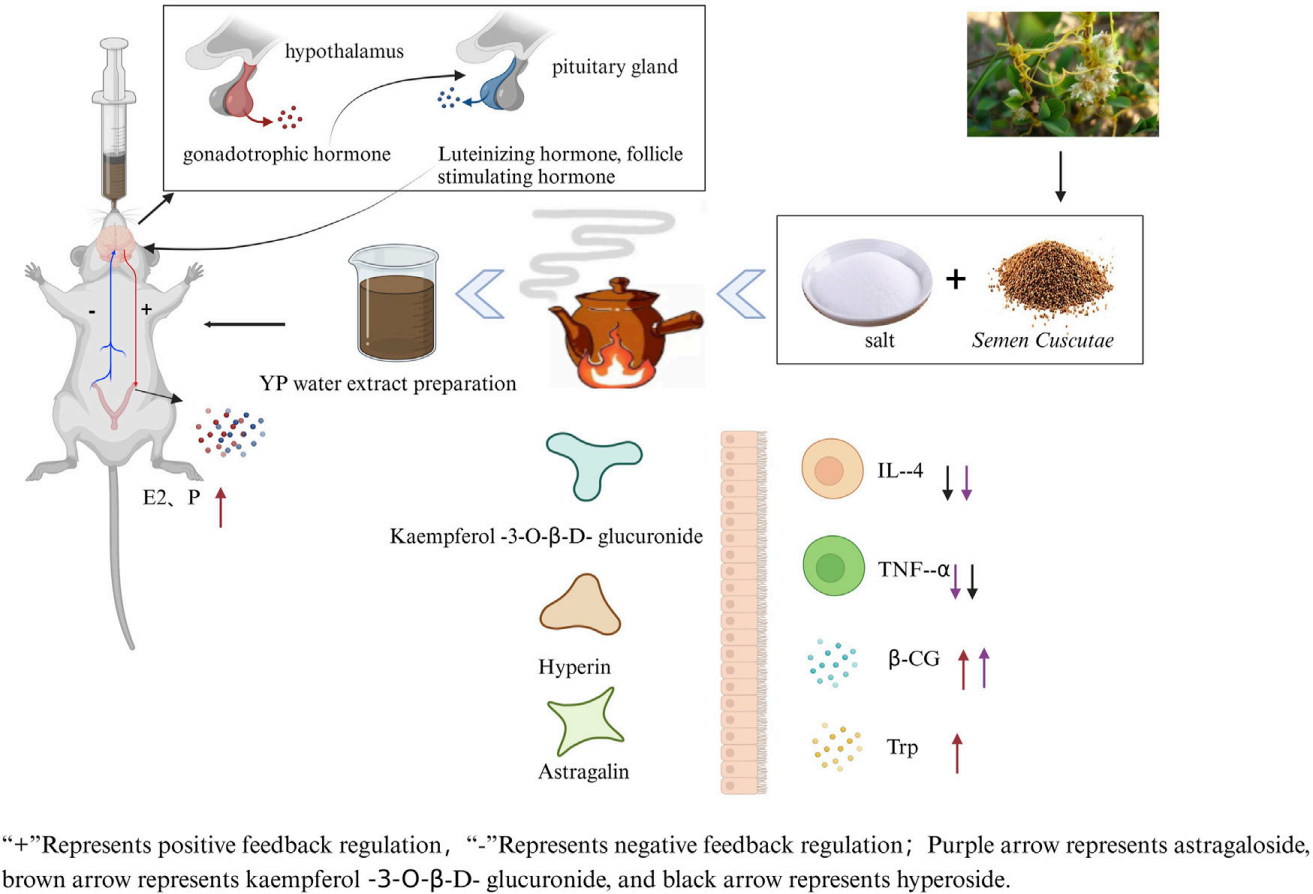
Mechanism of *Semen Cuscutae* salted on recurrent spontaneous abortion.

To further confirm whether YP can alleviate recurrent abortion induced by gavage of hydroxyurea combined with mifepristone by regulating serum biochemical indexes, the correlation between flavonoids and serum biochemical indexes was analyzed (Figure 4D). There was a significant positive correlation among KA, E2, and β-HCG (*p* < 0.05). However, AS and HY were negatively correlated with TNF-α (*p* < 0.05), and AS was positively correlated with β-HCG (*p* < 0.05). The mechanism of this study can be seen in Figure 6.

## Discussion

RSA is a complex disease caused by a variety of pathogenic factors. It is the most common complication of human pregnancy, with a high prevalence of approximately 10%–15% (Li et al., 2021a; Li et al., 2021b). β-HCG, P, and E2 can respond to embryonic development (Mi et al., 2021; Chen, 2018; Xiang, 2018). Other studies have shown that there is also a strong association between Th1/Th2 cytokines and the development of RSA. In this study, pregnant rats in the 5-, 7-, 9-, and 10-day groups all showed the following symptoms: rats curled up and hugged, dry and dull hair, thinning, weight loss, and sticky and loose stools in the anus. In addition, the longer it goes on, the more pronounced the symptoms become. After the abortifacient was given, there was bruising in the uterus after autopsy in the group on day 12, indicating the success of the RSA model. Studies have shown that when miscarriage occurs, maternal expression of Th1-type cytokines such as TNF-α is elevated, whereas the expression of Th2-type cytokines such as IL-4 was downregulated. This dysregulation of the immune homeostasis mediated by Th1- and Th2-type cytokines is one of the mechanisms leading to the occurrence of miscarriage (Kolanko et al., 2019). In the present study, we found an overall decreasing trend in Th1 type of inflammatory factor, which was elevated by the administration of the abortifacient. Meanwhile, Th2-type inflammatory factors were generally elevated. This suggests that YP can effectively regulate the levels of pregnancy hormones and Th1/Th2 cytokines in RSA. It corrects immune dysfunction, reduces miscarriage rates, and improves pregnancy outcomes. At the same time, P, E2, β-HCG, and TRP tended to increase with the duration of gestation and decreased with the administration of mifepristone. It has been found that β-HCG is secreted and produced by syncytiotrophoblast cells of the chorionic villi. The level of β-HCG increased exponentially in the first trimester of pregnancy, and then it decreased rapidly. Its main function is to stimulate the corpus luteum of pregnancy and secrete P and E2, which is beneficial for embryo development (Zhou, 2019), which is mutually confirmed by the results of this study. Tissue distribution experiments showed that AS could be detected in the heart, liver, spleen, lung, kidney, and uterus, indicating that AS could be widely distributed and metabolized in rats. Especially in the liver and kidney tissues, the concentration is higher, indicating that the kidney, liver, and uterus are the core target organs of the active ingredients of YP distribution. It also shows that YP can improve the distribution, absorption, and metabolism of KA in the body and promote its absorption in the kidney tissue, which is consistent with the traditional theory of “salt into the kidney channel,” while YP into the liver and kidney channel.

Transforming growth factor-β1 (TGF-β1) has been reported to be a secreted protein. It can induce inflammation in intrauterine adhesions (IUA) by enhancing the expression of pro-inflammatory factors such as TNF-α and IL-6 (Ai et al., 2020). HY has been shown to have anti-inflammatory pharmacological effects. In addition, as a main ingredient in Shou Zi Wan, it is widely used in the treatment of unexplained RSA. It is hypothesized that HY is closely related to uterine function, which coincides with the detection of chrysin in the uterus. Another study showed that kaempferol improves the doxorubicin-induced renal structure and function in rats by attenuating impairments in urine output, albumin/creatinine excretion, and creatinine clearance. It can also exert a nephroprotective effect by activating and upregulating SIRT 1, which in turn activates Nrf 2 and inhibits NF-κB p65 (Alagal et al., 2022). It can be hypothesized that KA has a close association with the kidneys, and therefore, this component can be detected in renal tissues.

In this study, we combined the results of gray correlation and Pearson correlation analyses to hypothesize that kaempferol, KA, AS, and HY may be the active ingredients of SP for the treatment of RSA. By reviewing the literature, the author found that kaempferol is an important flavonoid compound in S. It can promote primordial follicle activation and cell proliferation by regulating the PI3K/AKT signaling pathway. It also has the effect of promoting the diastolic effect of rat uterine smooth muscle (Santos et al., 2019). It provides theoretical support for the development of this ingredient for use in abortion-improving drugs. AS can upregulate the expression of ATP-binding transporter proteins A1 and G1 in macrophages. It promotes cholesterol efflux, inhibits inflammatory responses, and significantly reduces the secretion of inflammatory factors such as TNF-α and IL-6 (Zhao et al., 2021). HY inhibits macrophage chemotaxis, activates macrophages, and promotes macrophage differentiation from pro-inflammatory M1 subtype to anti-inflammatory M2 subtype. This mechanism is thought to be mediated by the direct regulation of NR4A1 by chrysosplenetin (Sun et al., 2021). Kaempferol has a significant inhibitory effect on COX1 and COX2. Once the cyclooxygenase pathway is activated, the membranes of the phospholipids rupture, resulting in the production of arachidonic acid, which is further converted via cyclooxygenase to prostaglandin analogs, causing inflammation. Thus, KA may prevent inflammation by inhibiting the cyclooxygenase pathway and thereby preventing inflammation. This is in keeping with the gray correlation results of this study, where all three active ingredients had a strong relationship with inflammatory factors. In addition, in angiogenesis mechanism studies, molecular docking results showed that kaempferol binds well to the target. Kaempferol promotes angiogenesis by reducing the reactive oxygen species (ROS) metabolism through the inhibition of the NF-κB pathway and upregulation of related transcriptional pathways (Imran et al., 2019). KA, HY, and AS are highly correlated with the estrogenic effects of total flavonoids from SS (Yue et al., 2023). This corroborates with the results of the present study. The results of this study provide data support and theoretical backing for further investigation of the efficacy and mechanism of these three components in the treatment of RSA.

It is innovative and feasible to investigate the molecular mechanisms of the three active components of *Semen Cuscutae*. In the future, mass spectrometry imaging technology will be used to pinpoint the target of action of KA, AS, HY, and proteomics, transteomics, transgenics, and other components of *Semen Cuscutae*. Furthermore, metabolomics will be used to study the three active ingredients from a multi-omics perspective. Relevant pathway proteins can be characterised through Western Blot experiment, and cellular experiment can be performed for the verification of the mechanism to explore the potential molecular mechanisms of the three active components of *Semen Cuscutae* to improve recurrent miscarriage using multi-omics technology.

## Conclusion

In conclusion, this paper mainly uses the correlation model of “body composition–efficacy index”, and we initially investigated the pharmacodynamics, pharmacokinetics and tissue distribution of HY, AS and KA in YP in the treatment of RSA. This research will help to further enrich the scientific connotation of the theory of the role of Chinese medicine processing excipients, and it has important guiding significance for the improvement of Chinese medicine quality standards and processing technology.

## Data Availability

The original contributions presented in the study are included in the article/Supplementary Material; further inquiries can be directed to the corresponding author.

## Supplementary material

SUPPLEMENTARY FIGURE S1 Network diagram of high-frequency drug association rules.

SUPPLEMENTARY FIGURE S2 Frequency of use of common herbs for the treatment of slippery fetus and fetal movement.

SUPPLEMENTARY FIGURE S3 HPLC characterization of 20 batches of *Semen Cuscutae* and salt-processed *Semen Cuscutae* tablets.

SUPPLEMENTARY FIGURE S4 Control characteristic profiles and HPLC plots of *Semen Cuscutae*, salt-processed *Semen Cuscutae*, and mixed controls.

SUPPLEMENTARY FIGURE S5 Micrograph of rat vaginal smear **(A)**: diestrus vaginal smear **(B)**: estrus vaginal smear (a large number of keratinised cells) **(C)**: diestrus vaginal smear (a small amount of keratinised cells) **(D)**, rat vaginal sperm smear. keratinised cells) **(C)**: diestrus vaginal smear (a small amount of keratinised cells) **(D)**, rat vaginal sperm smear.

SUPPLEMENTARY FIGURE S6 Typical chromatogram of index components and internal standard in the plasma and tissues of recurrent spontaneous abortion rat model. **(A)**: blank plasma; **(B)**: blank plasma+ astragaloside; **(C)**: blank plasma+ hyperoside; **(D)**: blank plasma+ kaempferol -3-O-β-D glucuronide; **(E)**: blank plasma+ internal standard; **(F)**: administration of plasma (astragaloside); **(G)**: administration of plasma (hyperin); **(H)**: administration of plasma (kaempferol -3-O-β-D -glucuronide); **(I)**: administration of plasma (internal standard); **(J)**: blank organization; **(K)**: blank tissue+ astragaloside; **(L)**: blank tissue+ hyperoside; **(M)**: blank tissue+ kaempferol-3-O-β-D-glucuronide; **(N)**: blank organization+ internal standard; **(O)**: administration organization (astragaloside); **(P)**: administration tissue (hyperin); **(Q)**: administration tissue (kaempferol-3-O-β-D-glucuronide); **(R)**: administration organization (internal standard).

## Abbreviations

AS, astragalin; AUC, area under curve; E2, estradiol; ELISA, enzyme-linked immunosorbent assay; ESI, electrospray ionization; HY, hyperin; IL-4, interleukin-4; IL-6, interleukin-6; IUA, intrauterine adhesions; KA, kaempferol-3-O-β-D-glucuronide; P, progesterone; QC, quality control samples; RSA, recurrent abortion; S, raw products of *Semen Cuscutae*; TGF-β1, transforming growth factor-β1; TNF-α, tumor necrosis factor-α; UHPLC-Q/TOF-MS, ultra-high performance liquid chromatography and quadrupole time-of-flight mass spectrometry; VEGF, vascular endothelial growth factor; YP, *Semen Cuscutae* processed with salt solution.

## References

[B1] Ai Y. Chen M. Liu J. Ren L. Yan X. Feng Y. (2020). lncRNA TUG1 promotes endometrial fibrosis and inflammation by sponging miR-590-5p to regulate Fasl in intrauterine adhesions. Int. Immunopharmacol. 86, 106703. 10.1016/j.intimp.2020.106703 32599321

[B2] Alagal R. I. Alfaris N. A. Alshammari G. M. Altamimi J. Z. Almousa L. A. Yahya M. A. (2022). Kaempferol attenuates doxorubicin-mediated nephropathy in rats by activating sirt1 signaling. J. Funct. Foods 89, 104918. 10.1016/j.jff.2021.104918

[B3] Chen R. X. (2018). The value of serum E2, progesterone and β-hCG levels in early pregnancy in pregnant women with preeclamptic miscarriage in predicting pregnancy outcome. Maternal Child Health China. 33, 1371–1373.

[B4] Deng T. Q. Liao X. Y. Zhu S. M. (2022). Recent advances in treatment of recurrent spontaneous abortion. Obstet. Gynecol. Surv. 77 (6), 355–366. 10.1097/OGX.0000000000001033 35672876 PMC9169761

[B5] Huang Y. Yu H. Y. Xiong J. W. Ding X. M. Xu B. Y. Liu L. N. (2022). Chemical profiling of raw product of Semen Cuscuta and stir-baking Semen Cuscuta with salt solution based on UHPLC-Q/TOF-MS combined multivariate statistical analysis. Chin. J. New Drugs 31 (15).

[B6] Imran M. Salehi B. Sharifi-Rad J. Aslam Gondal T. Saeed F. Imran A. (2019). Kaempferol: a key emphasis to its anticancer potential. Molecules 24, 2277. 10.3390/molecules24122277 31248102 PMC6631472

[B7] Kolanko E. Kopaczka K. Koryciak-Komarska H. Czech E. Szmytkowska P. Gramignoli R. (2019). Increased immunomodulatory capacity of human amniotic cells after activation by pro-inflammatory chemokines. Eur. J. Pharmacol. 859, 172545. 10.1016/j.ejphar.2019.172545 31319066

[B8] La X. L. Wang W. J. Zhang M. Liang L. (2021). Definition and multiple factors of recurrent spontaneous abortion. Adv. Exp. Med. Biol. 1300, 231–257. 10.1007/978-981-33-4187-6_11 33523437

[B9] Li D. Zheng L. Zhao D. Xu Y. Wang Y. (2021a). The role of immune cells in recurrent spontaneous abortion. Reprod. Sci. 28 (12), 3303–3315. 10.1007/s43032-021-00599-y 34101149 PMC8186021

[B10] Li J. Feng D. He S. Wu Q. Su Z. Ye H. (2021b). Meta-analysis: association of homocysteine with recurrent spontaneous abortion. Women Health 61, 713–720. 10.1080/03630242.2021.1957747 34334120

[B11] Liu Y. Liu Y. Ding W. Wang X. L. (2021). Advances in research of pharmacologic action of Cuscuta chinensis tonifying the kidney. J. Yichun Univ. 43 (09), 22–25.

[B12] Mi Y. R. Li X. Q. You J. P. Zhe N. (2021). Effect of solidifying and stabilizing fetal soup combined with progesterone on serum sex hormones and MCP-1 and IL-1β levels in patients with preeclampsia. New Chin. Med. Clin. Pharmacol. 32, 123–127.

[B13] Santos J. M. S. Monte A. P. O. Lins T. L. B. G. Barberino R. S. Menezes V. G. Gouveia B. B. (2019). Kaempferol can be used as the single antioxidant in the *in vitro* culture medium, stimulating sheep secondary follicle development through the phosphatidylinositol 3-kinase signaling pathway. Theriogenology 136, 86–94. 10.1016/j.theriogenology.2019.06.036 31254726

[B14] Su L. L. Tong H. J. Zhang J. B. Hao M. Fei C. H. Ji D. (2022). Revealing the mechanism of raw and vinegar-processed Curcuma aromatica Salisb. [Zingiberaceae] regulates primary dysmenorrhea in rats via integrated metabolomics. Front. Pharmacol. 13, 926291. 10.3389/fphar.2022.926291 36176430 PMC9513393

[B15] Sun K. Luo J. Jing X. Xiang W. Guo J. Yao X. (2021). Hyperoside ameliorates the progression of osteoarthritis: an *in vitro* and *in vivo* study. Phytomedicine 80, 153387. 10.1016/j.phymed.2020.153387 33130473

[B16] Wang X. L. Gao H. Y. Tan S. Xu C. Xu F. Q. Wang T. S. (2021). An integrated approach to uncover quality markers of stir-baking Semen Cuscuta with salt solution preventing recurrent spontaneous abortion based on chemical and metabolomic profiling. J. Chromatogr. B Anal. Technol. Biomed. Life Sci. 1177, 122727. 10.1016/j.jchromb.2021.122727 34102535

[B17] Wang X. L. Jiang Y. Fan L. L. Wu D. L. Han Y. Q. Jin C. S. (2020). Modern research on theory of “salt-processing enhancing drug into kidney meridian” proposed by healer Jia-mo Chen from Xinan. Chin. Tradit. Herb. Drugs 51 (05), 1336–1342.

[B18] Xiang H. (2018). Clinical significance of β-hCG, PROG, E2 and CA125 in patients with preeclampsia. China Maternal Child Health 33, 2546–2549.

[B19] Xu B. Y. Ding X. M. Jin C. S. Wu D. L. Peng T. Y. Wang X. L. (2023a). Chromatogram of Chinese dodder seed unprocessedor processed with salt solutionon high-performance liquid chromatography and content determination of five main component. J. Anhuiuniv Chin. Med. 42 (02), 95–100.

[B20] Xu B. Y. Yang Z. T. Zhang X. Liu Z. L. Huang Y. Ding X. M. (2023b). 16S rDNA Sequencing combined with metabolomics profiling with multi-index scoring method reveals the mechanism of salt-processed Semen Cuscuta in Bushen Antai mixture on kidney yang deficiency syndrome. J. Chromatogr. B Anal. Technol. Biomed. Life Sci. 1216, 123602. 10.1016/j.jchromb.2023.123602 36652816

[B21] Xu Y. J. Ren X. L. Zeng Q. (2023c). Experience in treating fetal leakage and fetal movement of spleen and kidney weakness of traditional Chinese medicine. Chin. J. Ethnomed Ethnopharm 32 (15), 89–92+100.

[B22] Yue X. Song H. Xu Y. Liu D. S. Sun X. M. Li W. L. (2023). Screening of active ingredients for estrogenic effects in total flavonoids of Cuscuta chinensis. Chin. Pharm. 34, 569–574.

[B23] Zhang X. Huang Y. Yang Z. T. Xu B. Liu Z. L. Ding X. M. (2024). Exploring the mechanism of Semen Cuscutae processed with salt solution in improving kidney deficiency miscarriage based on serum pharmacochemistry and network pharmacology. Arabian J. Chem. 17 (2024), 105456. 10.1016/j.arabjc.2023.105456

[B24] Zhao Z. W. Zhang M. Wang G. Zou J. Gao J. H. Zhou L. (2021). Astragalin retards atherosclerosis by promoting cholesterol efflux and inhibiting the inflammatory response via upregulating ABCA1 and ABCG1 expression in macrophages. J. Cardiovasc Pharmacol. 77, 217–227. 10.1097/FJC.0000000000000944 33165140

[B25] Zhou S. M. (2019). Study on the prognostic value of serum CA125, HCG and progesterone in predicting early preeclampsia. Chin. J. Mod. Med. 21, 84–85.

