# Supplementary figures and images for: Study on pharmacokinetic and tissue distribution of hyperin, astragalin, kaempferol-3-O-β-D-glucuronide from rats with multiple administrations of Semen Cuscutae processed with salt solution with effect of treating recurrent spontaneous abortion
Source: Front Pharmacol. 2024 Sep 16;15:1440810. doi: 10.3389/fphar.2024.1440810 (PMC11439818; doi:10.3389/fphar.2024.1440810)

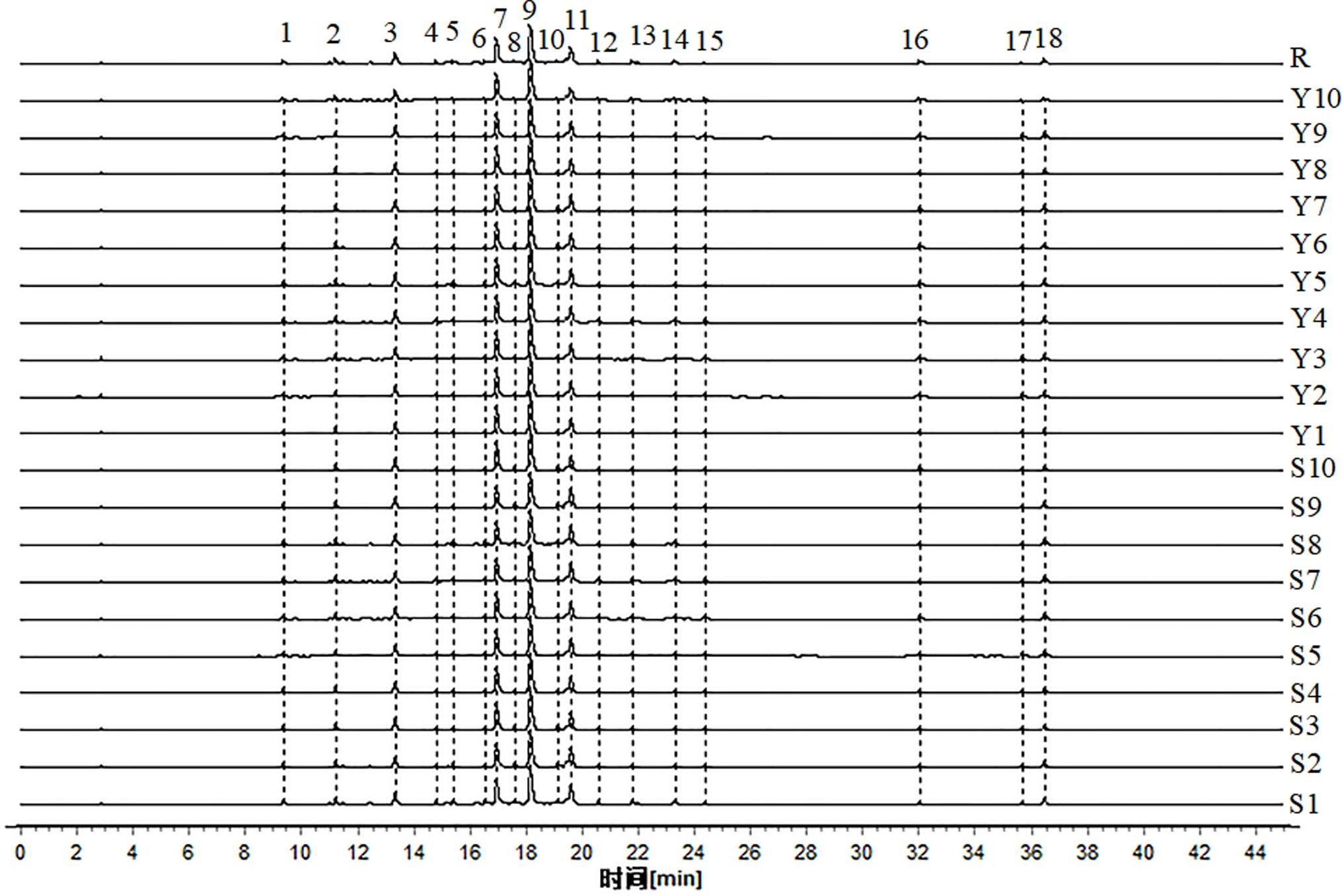

Supplement: Supplementary file 1 [file Image3.JPEG]

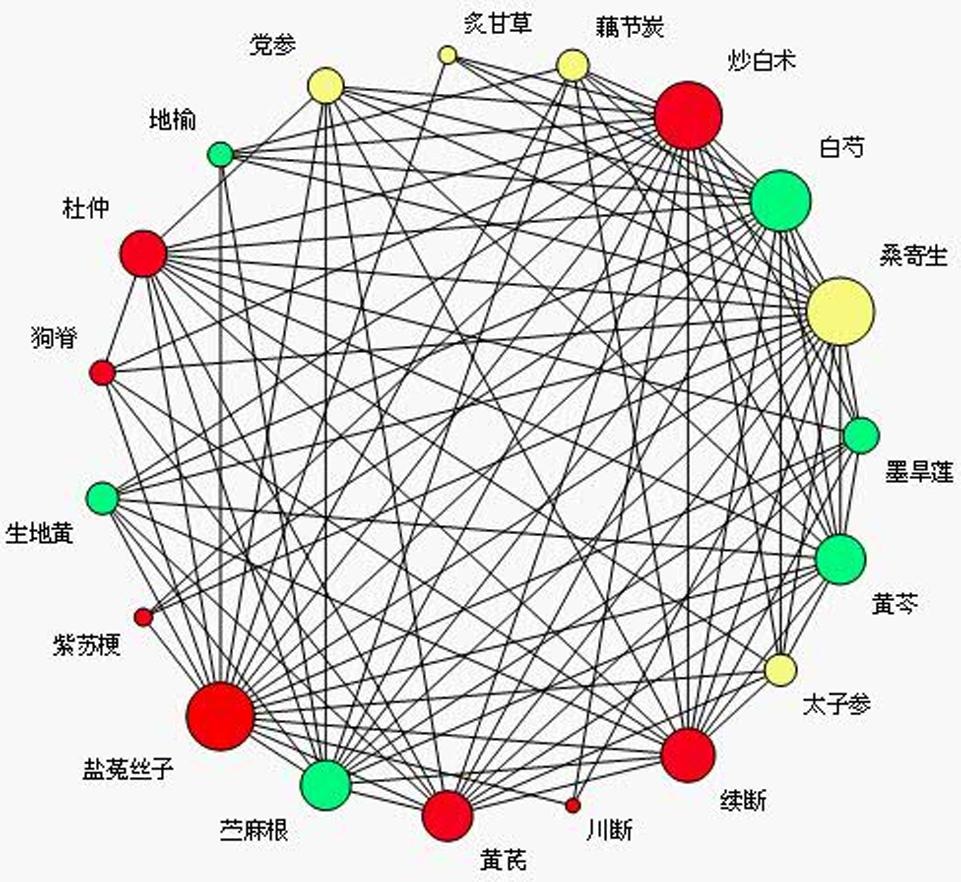

Supplement: Supplementary file 3 [file Image1.JPEG]

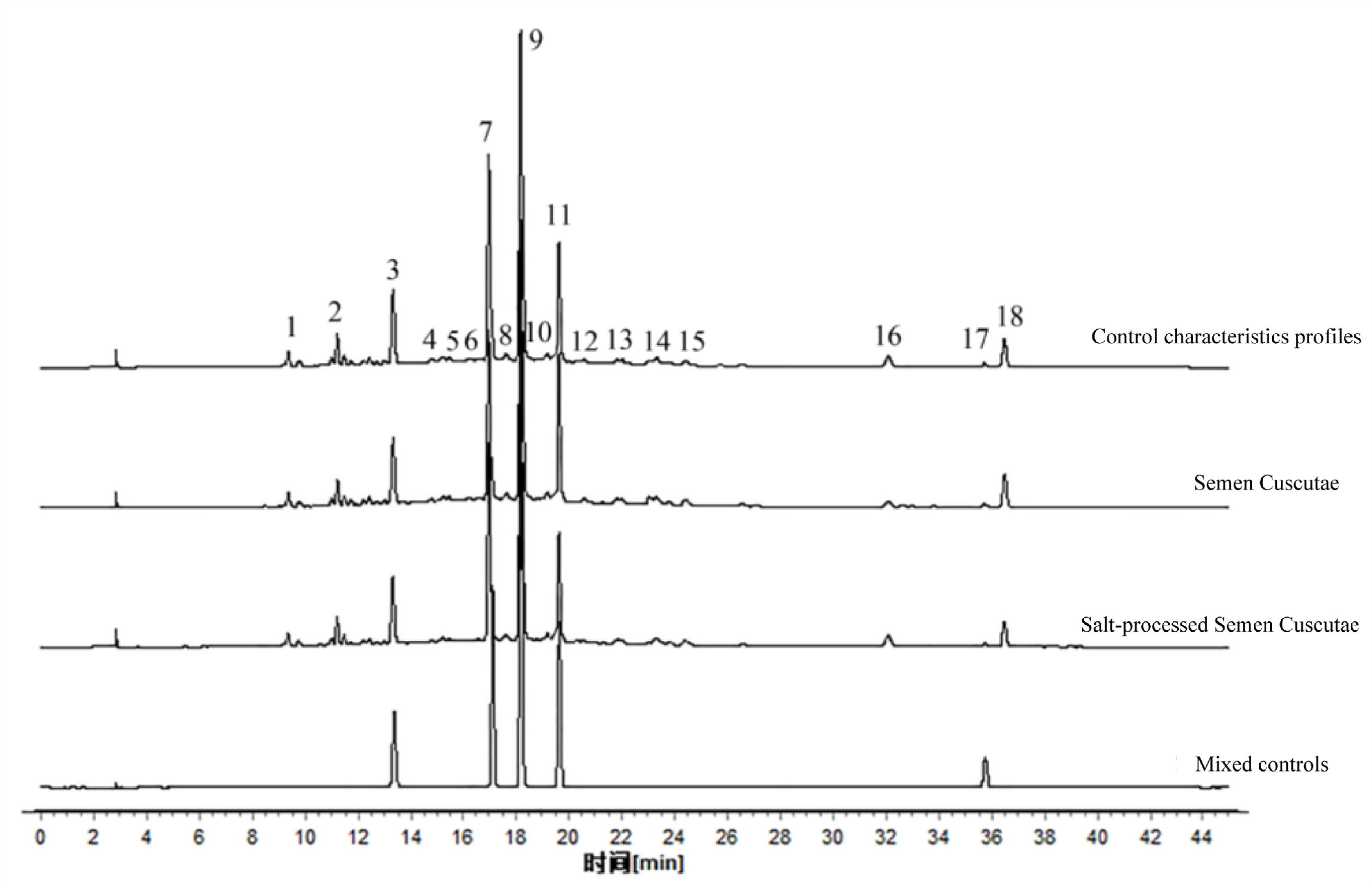

Supplement: Supplementary file 4 [file Image4.JPEG]

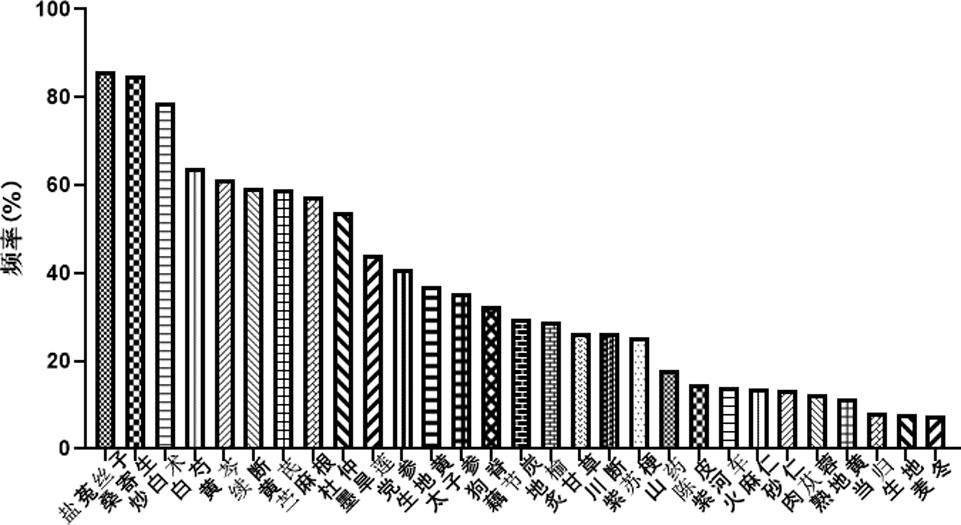

Supplement: Supplementary file 5 [file Image2.JPEG]

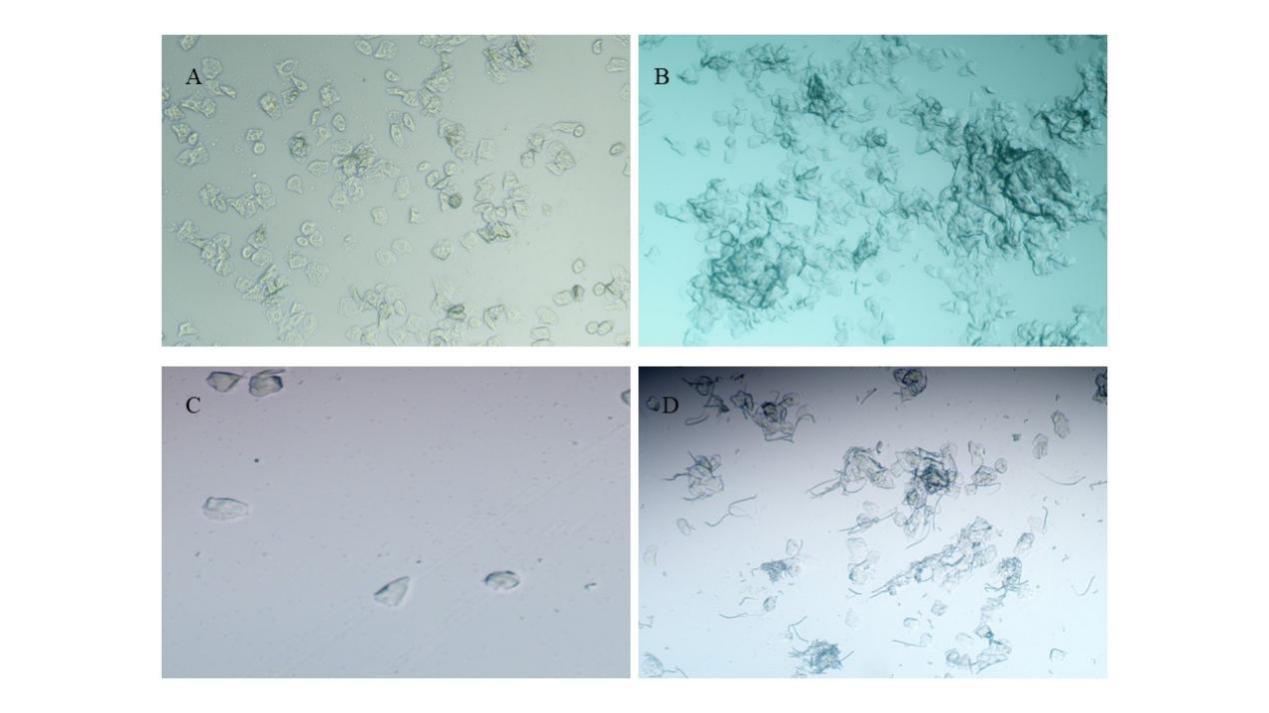

Supplement: Supplementary file 6 [file Image5.JPEG]

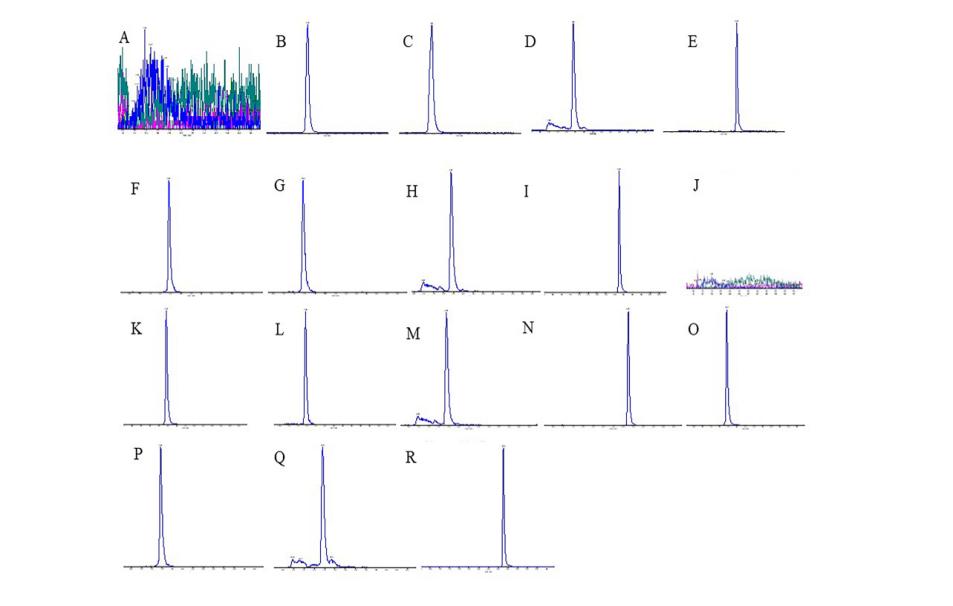

Supplement: Supplementary file 7 [file Image6.JPEG]
