# Supplementary material for: Study on pharmacokinetic and tissue distribution of hyperin, astragalin, kaempferol-3-O-β-D-glucuronide from rats with multiple administrations of Semen Cuscutae processed with salt solution with effect of treating recurrent spontaneous abortion
Source: Front Pharmacol. 2024 Sep 16;15:1440810. doi: 10.3389/fphar.2024.1440810 (PMC11439818; doi:10.3389/fphar.2024.1440810)
Supplement: Supplementary file 2 [file Table1.DOCX]

| Compounds | Plasma | Standard curve equation | R^2^ | LLOQ /(ng) | linearity range/(ng) |
| --- | --- | --- | --- | --- | --- |
| Astragalin | plasma | y=0.02233x+0.02783 | 0.9998 | 1 | 1-1000 |
|  | liver | y = 0.0577x + 0.2656 | 0.9972 | 1 | 1-500 |
|  | heart | y = 0.0568x + 0.3119 | 0.9954 | 1 | 1-500 |
|  | spleen | y = 0.0476x + 0.0046 | 0.9957 | 1 | 1-500 |
|  | lung | y = 0.0562x - 0.1523 | 0.9974 | 1 | 1-500 |
|  | kidney | y = 0.0573x - 0.391 | 0.9981 | 1 | 1-500 |
|  | womb | y = 0.0464x + 0.5365 | 0.9965 | 1 | 1-500 |
| Kaempferol -3-O -β-D-glucuronide | plasma | y=0.02575x+0.00281 | 0.9998 | 1 | 1-1000 |
|  | kidney | y = 0.0346x + 0.1612 | 0.9942 | 1 | 1-500 |
| Hyperin | plasma | y=0.01109x+0.01981 | 0.9996 | 1 | 1-1000 |
|  | womb | y = 0.0493x + 0.1368 | 0.9967 | 1 | 1-500 |

Table 1 The standard curve ranges and LLOQ of 3 representative components in plasma and tissues

Table 2 The precision and accuracy of 3 representative components in plasma and tissues（*n*=6）

| Compounds | Plasma/tissue | Nominal Concentration | RE/% | | RSD/% | |
| --- | --- | --- | --- | --- | --- | --- |
|  |  |  | inter-day | Intra-day | inter-day | Intra-day |
| Astragalin | plasma | 2.5 | 1.97 | -4.06 | 7.11 | 14.22 |
|  |  | 100 | -5.15 | 10.58 | 8.24 | 11.25 |
|  |  | 800 | -5.02 | -11.93 | 6.68 | 9.52 |
|  | liver | 2.5 | -1.73 | -5.80 | 7.32 | 14.32 |
|  |  | 50 | 7.32 | -1.58 | 10.77 | 8.82 |
|  |  | 400 | 14.32 | 3.59 | 8.93 | 7.43 |
|  | heart | 2.5 | -0.27 | 4.16 | 4.96 | 11.90 |
|  |  | 50 | 10.67 | 5.88 | 12.93 | 10.91 |
|  |  | 400 | 0.77 | 3.06 | 8.91 | 8.24 |
|  | spleen | 2.5 | -0.33 | -6.02 | 4.36 | 8.20 |
|  |  | 50 | 5.43 | 3.08 | 14.08 | 10.45 |
|  |  | 400 | -0.80 | -0.18 | 10.07 | 8.10 |
|  | lung | 2.5 | -0.33 | 2.82 | 3.60 | 6.31 |
|  |  | 50 | -6.67 | -1.26 | 7.56 | 11.48 |
|  |  | 400 | 1.37 | -1.17 | 7.38 | 7.73 |
|  | kidney | 2.5 | 1.53 | 0.82 | 5.10 | 5.33 |
|  |  | 50 | -9.40 | -4.76 | 5.11 | 7.23 |
|  |  | 400 | 5.70 | 3.73 | 8.18 | 10.19 |
|  | womb | 2.5 | -9.80 | -9.93 | 11.90 | 12.61 |
|  |  | 50 | 0.43 | -1.77 | 4.48 | 6.69 |
|  |  | 400 | -2.56 | 3.33 | 4.52 | 9.94 |
| Hyperin | plasma | 2.5 | 11.77 | -4.93 | 7.07 | 12.39 |
|  |  | 100 | -0.34 | -4.64 | 9.96 | 12.09 |
|  |  | 800 | -8.56 | -14.13 | 9.32 | 9.42 |
|  | womb | 2.5 | 3.60 | 9.16 | 8.76 | 13.14 |
|  |  | 50 | -2.50 | -0.78 | 11.11 | 10.05 |
|  |  | 400 | 3.90 | 5.48 | 5.80 | 9.16 |
| Kaempferol-3-O-β-D- glucuronide | plasma | 2.5 | -4.87 | -14.38 | 6.04 | 10.72 |
|  |  | 100 | 10.32 | 3.21 | 10.12 | 10.29 |
|  |  | 800 | -5.69 | -13.26 | 7.88 | 7.05 |
|  | kidney | 2.5 | -11.20 | -12.89 | 14.65 | 14.72 |
|  |  | 50 | -5.50 | -1.87 | 7.56 | 6.77 |
|  |  | 400 | -0.69 | 6.96 | 3.64 | 8.61 |

Table3 The extraction recovery and matrix effects of 3 representative components in plasma and

tissues（*n*=6）

| Compounds | Plasma | | Nominal Concentration | | Extraction  Recovery | | | | Matrix effect | | | |  |
| --- | --- | --- | --- | --- | --- | --- | --- | --- | --- | --- | --- | --- | --- |
|  |  |  |  |  | Mean±SD | | RSD，% | | Mean±SD | | RSD，% | |  |
| Astragalin | plasma | | 2.5 | | 97.93±5.88 | | 6.00 | | 93.79±11.51 | | 12.28 | |  |
|  |  |  | 100 | | 98.71±3.65 | | 3.70 | | 92.69±2.70 | | 2.90 | |  |
|  |  |  | 800 | | 94.08±3.91 | | 4.16 | | 94.00±5.92 | | 6.29 | |  |
|  | liver | | 2.5 | | 93.80±6.92 | | 7.38 | | 96.40±9.74 | | 10.10 | |  |
|  |  |  | 50 | | 92.29±7.44 | | 8.06 | | 97.65±8.33 | | 8.53 | |  |
|  |  |  | 400 | | 100.99±9.68 | | 9.58 | | 97.34±14.46 | | 14.85 | |  |
|  | heart | | 2.5 | | 97.58±9.19 | | 9.41 | | 107.21±8.01 | | 7.47 | |  |
|  |  |  | 50 | | 93.08±5.66 | | 6.08 | | 106.35±9.55 | | 8.98 | |  |
|  |  |  | 400 | | 97.29±1.70 | | 1.75 | | 99.37±8.72 | | 8.77 | |  |
|  | spleen | | 2.5 | | 104.13±7.96 | | 7.64 | | 105.75±7.81 | | 7.39 | |  |
|  |  |  | 50 | | 94.78±9.82 | | 10.36 | | 101.82±7.55 | | 7.41 | |  |
|  |  |  | 400 | | 93.49±5.18 | | 5.54 | | 104.43±3.67 | | 3.52 | |  |
|  | lung | | 2.5 | | 95.60±8.03 | | 8.40 | | 95.99±10.92 | | 11.37 | |  |
|  |  |  | 50 | | 93.22±4.98 | | 5.34 | | 95.72±8.18 | | 10.83 | |  |
|  |  |  | 400 | | 91.96±7.10 | | 7.75 | | 99.64±3.67 | | 3.68 | |  |
|  | kidney | | 2.5 | | 94.29±4.70 | | 4.99 | | 97.72±8.18 | | 8.37 | |  |
|  |  |  | 50 | | 102.00±9.05 | | 8.87 | | 96.96±9.58 | | 9.88 | |  |
|  |  |  | 400 | | 94.86±6.33 | | 6.67 | | 97.37±7.19 | | 7.38 | |  |
|  | womb | | 2.5 | | 97.54±5.38 | | 5.51 | | 102.25±6.74 | | 6.59 | |  |
|  |  |  | 50 | | 98.55±7.94 | | 8.06 | | 102.49±6.91 | | 6.74 | |  |
| Hyperin | plasma | | 400 | | 97.45±9.88 | | 10.14 | | 95.43±10.15 | | 10.64 | |  |
|  |  |  | 2.5 | | 98.92±7.46 | | 7.54 | | 91.84±4.85 | | 5.29 | |  |
|  |  |  | 100 | | 97.02±1.43 | | 1.47 | | 92.30±3.17 | | 3.44 | |  |
|  |  |  | 800 | | 92.71±4.79 | | 5.16 | | 96.13±3.32 | | 3.45 | |  |
|  | womb | | 2.5 | | 101.33±3.48 | | 3.44 | | 96.19±11.14 | | 11.57 | |  |
|  |  |  | 50 | | 100.46±4.13 | | 4.11 | | 96.81±9.43 | | 9.74 | |  |
|  |  |  | 400 | | 95.67±7.72 | | 8.07 | | 100.43±4.37 | | 4.35 | |  |
| Kaempferol-3-O-β-D- glucuronide | | plasma | | 2.5 | | 96.08±7.39 | | 7.70 | | 95.15±3.55 | | 3.74 | |
|  |  |  |  | 100 | | 102.92±4.38 | | 4.26 | | 92.63±3.12 | | 3.37 | |
|  |  |  |  | 800 | | 104.96±6.52 | | 6.21 | | 99.03±2.75 | | 2.78 | |
|  |  | kidney | | 2.5 | | 91.89±2.28 | | 2.48 | | 98.60±6.18 | | 6.27 | |
|  |  |  |  | 50 | | 90.87±7.74 | | 8.52 | | 98.91±9.66 | | 9.76 | |
|  |  |  |  | 400 | | 94.40±2.87 | | 3.04 | | 97.00±7.15 | | 7.37 | |

Table 4 Study on the stability of three representative components in plasma and tissues(*n*＝6)

|  | | | Measured concentration | | | | | |
| --- | --- | --- | --- | --- | --- | --- | --- | --- |
| Compounds | Plasma | Nominal Concentration | Short-term (room temperature, 12 h) | | Long-term (−20 ◦C, 4  weeks) | | Three freeze-thaw  cycles | |
|  | | | Mean±SD | RSD  (%) | Mean±SD | RSD  (%) | Mean±SD | RSD  (%) |
| Astragalin | Plasma | 2.5 | 2.58±0.30 | 11.83 | 2.81±0.395 | 14.04 | 2.75±0.35 | 12.81 |
|  |  | 100 | 104.29±10.89 | 10.45 | 103.22±8.77 | 8.50 | 105.74±7.16 | 6.77 |
|  |  | 800 | 701.97±62.81 | 8.95 | 739.67±80.17 | 10.84 | 742.73±67.87 | 9.14 |
|  | liver | 2.5 | 2.48±0.20 | 8.24 | 2.67±0.17 | 6.62 | 2.82±0.17 | 6.07 |
|  |  | 50 | 50.77±4.58 | 9.02 | 48.61±5.31 | 10.92 | 52.70±5.04 | 9.55 |
|  |  | 400 | 418.65±37.37 | 8.93 | 407.50±27.72 | 6.80 | 415.03±29.99 | 7.23 |
|  | heart | 2.5 | 2.76±0.38 | 13.64 | 2.36±0.27 | 11.29 | 2.8±0.08 | 2.79 |
|  |  | 50 | 54.77±5.42 | 9.90 | 53.13±2.20 | 4.15 | 43.03±2.64 | 6.14 |
|  |  | 400 | 398.33±20.33 | 5.10 | 403.17±38.63 | 9.58 | 394.83±20.95 | 5.31 |
| Astragalin | spleen | 2.5 | 2.67±0.18 | 6.62 | 2.47±0.28 | 11.21 | 2.41±0.32 | 11.35 |
|  |  | 50 | 48.61±5.31 | 10.92 | 52.70±5.04 | 9.55 | 48.07±4.09 | 8.52 |
|  |  | 400 | 407.50±27.72 | 6.80 | 415.03±29.99 | 7.23 | 425.00±36.56 | 8.60 |
|  | Lung | 2.5 | 2.40±0.18 | 7.33 | 2.37±0.18 | 7.73 | 2.63±0.34 | 12.99 |
|  |  | 50 | 53.17±5.20 | 5.79 | 49.82±5.60 | 11.23 | 54.60±7.19 | 13.16 |
|  |  | 400 | 430.18±24.51 | 5.70 | 426.27±53.24 | 12.49 | 387.55±20.16 | 5.20 |
|  | kidney | 2.5 | 2.47±0.21 | 8.68 | 2.63±0.16 | 6.09 | 2.54±0.11 | 4.45 |
|  |  | 50 | 49.62±5.42 | 10.92 | 47.18±5.76 | 12.21 | 47.78±2.96 | 6.20 |
|  |  | 400 | 412.47±25.43 | 6.17 | 404.50±32.02 | 7.92 | 417.67±31.27 | 7.45 |
|  | womb | 2.5 | 2.40±0.18 | 11.56 | 2.37±0.18 | 14.94 | 2.63±0.34 | 8.21 |
|  |  | 50 | 53.17±5.20 | 11.01 | 49.82±5.60 | 13.05 | 54.60±7.19 | 11.51 |
|  |  | 400 | 430.18±24.51 | 10.65 | 426.27±53.24 | 7.78 | 387.55±20.16 | 14.25 |
| Hyperin | plasma | 2.5 | 2.22±0.19 | 8.44 | 2.40±0.32 | 13.35 | 2.20±0.31 | 14.17 |
|  |  | 100 | 86.22±8.44 | 9.79 | 87.08±5.81 | 6.68 | 87.39±5.59 | 6.40 |
|  |  | 800 | 697.10±46.69 | 6.70 | 683.50±43.70 | 6.40 | 685.80±15.19 | 2.21 |
|  | womb | 2.5 | 2.81±0.40 | 14.51 | 2.60±0.28 | 10.82 | 2.77±0.32 | 11.68 |
|  |  | 50 | 49.18±4.33 | 8.81 | 50.30±4.24 | 8.42 | 49.88±5.59 | 11.20 |
|  |  | 400 | 397.37±56.63 | 14.25 | 411.67±27.09 | 6.58 | 413.83±23.58 | 5.70 |
| Kaempferol-3-O-β-D- glucuronide | plasma | 2.5 | 2.16±0.17 | 7.74 | 2.18±0.27 | 12.36 | 2.19±0.20 | 8.96 |
|  |  | 100 | 97.38±8.71 | 8.94 | 93.49±8.30 | 8.88 | 92.77±4.83 | 5.21 |
|  |  | 800 | 685.92±57.77 | 8.42 | 706.73±28.45 | 4.03 | 714.27±20.54 | 2.88 |
|  | kidney | 2.5 | 2.25±0.24 | 10.66 | 2.78±0.22 | 7.93 | 2.67±0.18 | 6.62 |
|  |  | 50 | 43.24±6.29 | 14.56 | 51.32±6.50 | 12.68 | 44.62±5.19 | 11.64 |
|  |  | 400 | 395.25±29.50 | 7.46 | 385.33±31.07 | 8.06 | 399.83±20.66 | 5.17 |
